# Hepatitis C elimination: challenges with under-diagnosis and under-treatment

**DOI:** 10.12688/f1000research.15892.1

**Published:** 2019-01-14

**Authors:** Norah A Terrault

**Affiliations:** 1 Division of Gastroenterology & Hepatology, University of California, San Francisco, San Francisco, CA, USA

**Keywords:** baby boomers, PWIDs, direct-acting antivirals, screening, cure

## Abstract

Hepatitis C infection has affected 189 million people globally and more than 4 million in the US. Owing to remarkable advances in the therapeutic sphere, essentially all infected patients can be expected to achieve cure. This provides an unprecedented opportunity to eliminate the risk of complications from hepatitis C and to reduce the spread of the virus to others. To achieve this, a streamlined cascade of care from diagnosis to treatment may be enacted. Although great strides have been made, under-diagnosis and under-treatment remain major hurdles.

## Introduction

**Figure 1.**
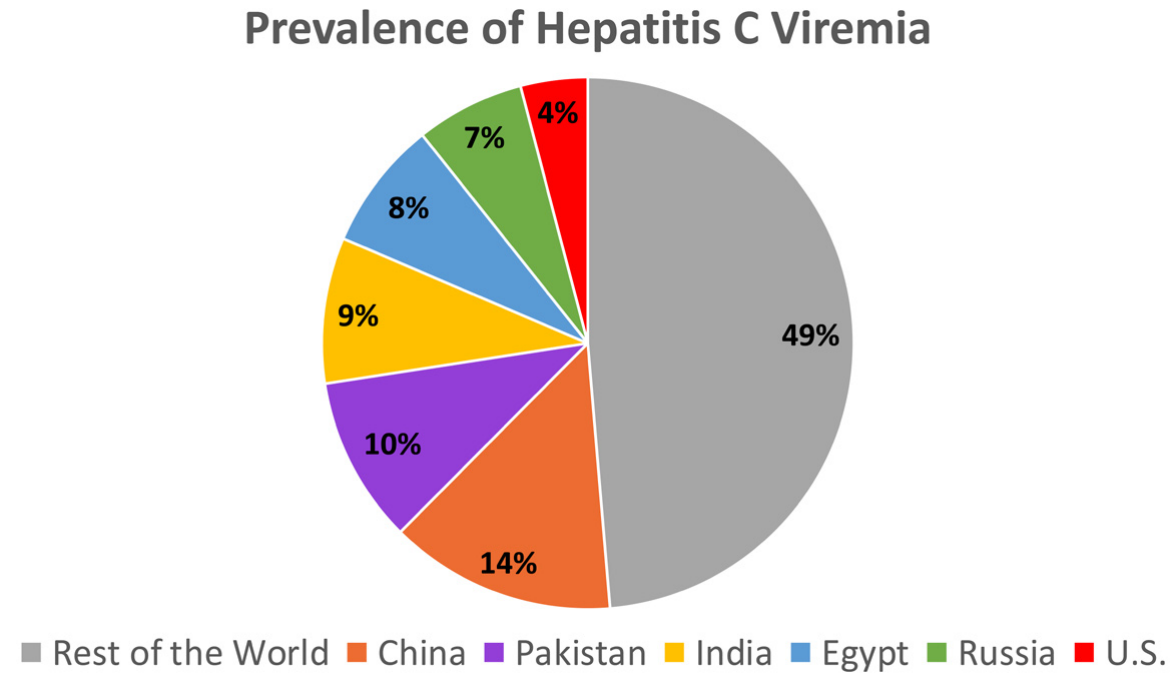
Countries with a higher burden of chronic hepatitis C virus (HCV). Twenty-eight countries account for 80% of viremic HCV infections. Six countries carry 50% of the global hepatitis C burden: China, Pakistan, India, Egypt, Russia, and the US ^3^.

Hepatitis C virus (HCV) is a major cause of cirrhosis, liver cancer, and mortality worldwide ^1^. Spread via contaminated blood or blood-contaminated objects, blood transfusions, or contact with unsterile needles (via injection drug use and medical care) has been the prime driver of HCV transmission around the globe. Once infected, the majority will remain chronically infected unless treatment is provided. After several decades of living with the chronic infection, a substantial proportion will come to suffer the consequences of cirrhosis and liver cancer. Additionally, HCV has effects beyond the liver, and extrahepatic complications are as diverse as diabetes, lymphoma, and chronic kidney disease ^2^. Recent estimates indicate that at least 71 million people have chronic HCV viremia globally ^3^, whereas a prior estimate (2010) was 80 million ^4^; this decline is related in part to improved epidemiologic data from some countries but also reflects deaths due to liver complications among those with chronic HCV. An estimated 500,000 deaths from HCV occurred in 2010 ^5^, and whereas in some sectors mortality rates are decreasing, the global trend in deaths is still increasing ^6^. Unique challenges are present in different countries. Six countries carry 50% of the global HCV burden: China, Pakistan, India, Egypt, Russia, and the US ( Figure 1) ^3, 7^. Reducing disease burden in every country requires not only enhanced rates of diagnosis and treatment but also strategies to prevent new infections, and countries with high (>3%) per-capita prevalence—such as Pakistan, Russia, Mongolia, Egypt, and Georgia—are the most challenged in meeting elimination goals.

Despite these rather sobering statistics, this is a time of great optimism. Direct-acting antiviral (DAA) drugs provide a simple, well-tolerated, and highly effective treatment, and cure rates are approaching 100% in adherent patients. In 2015, an estimated 700,000 persons with chronic HCV—about 1% of the total infected population—achieved eradication of their HCV infection ^8^. The World Health Organization (WHO) has called for elimination of HCV——and for reductions in HCV-associated mortality by 65% and in the rate of new infections by 90% by 2030. It is argued that elimination without the availability of a vaccine has never been attained for any infectious disease ^9^ and that a better goal might be “control” rather than elimination. Regardless, we have the capacity to diagnose and cure a chronic condition that is associated with significant morbidity and mortality, so the goal should be to reach as many infected persons as possible. Moreover, since HCV transmission requires blood––blood contact and is inefficiently transmitted by sexual or household transmission (with few exceptions), harm reduction strategies targeting major routes of transmission are highly feasible. In this review, the focus is on under-diagnosis and under-treatment as the primary culprits undermining the US goals of HCV elimination.

## Under-diagnosis: the largest gap in the cascade of care

**Figure 2.**
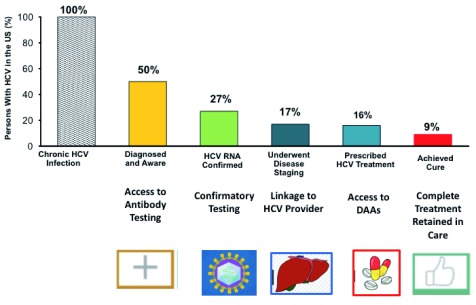
Hepatitis C virus (HCV) cascade of care. Of the 3.5 million Americans estimated to be infected with HCV, 50% have undergone anti-HCV testing, the first step in the cascade of care. Next, confirmation testing for viremia (HCV RNA testing) is needed. Once infection is confirmed, linkage with a provider who is expert in HCV treatment is needed (primary care or specialist) and additioal steps include testing for HCV genotype and staging of liver disease. Once treatment is prescribed, there are additional steps to get the medication approved and the patient to complete the treatment. As shown, there are multiple points along the cascade of care where interruption can occur, leading to decreased numbers of persons achieving HCV cure ^31^. Current HCV elimination efforts are focused on reducing gaps along the cascade of care.

An estimated 1.8 million Americans remain unaware of their HCV infection ^10^. Prior to 2012, screening for HCV was based on the presence of risk factors for HCV acquisition. However, this approach was unsuccessful for multiple reasons. First, clinicians lacked the knowledge or time (or both) to question patients on those risk factors ( Table 1); second, some patients did not wish to disclose risk factors; and, third, risk-based screening did not capture all infected persons. For example, the re-use of needles and syringes without adequate sterilization in the context of medical care in many developing and transitional countries is a key source of HCV transmission ^11, 12^, yet iatrogenic exposure from medical care in countries with moderate to high endemicity of HCV is not listed among the indications for HCV screening in the US. Adopting an approach similar to that used for hepatitis B, where individuals originating from countries of high HCV endemicity are screened for HCV, may increase case identification rates, especially among the foreign-born. An important change made to screening occurred in 2012, when the Centers for Disease Control and Prevention (CDC) ^13^ and the US Preventive Services Task Force (USPSTF) recommended one-time HCV testing for the 1945––1965 birth cohort. US prevalence studies identified these “baby boomers” as having the highest prevalence of HCV (50% of all infections), making this group a rich source for HCV case finding. Indeed, this addition to the screening recommendations is likely responsible for the reduction in undiagnosed HCV in the US from 70 to 50% that has been achieved in the past five years ^14^. However, to meet the 90% diagnosed target by 2030, the targeted rate of diagnosis should be 110,000 per year until 2020, 89,000 per year between 2020 and 2024, and more than 70,000 per year between 2025 and 2030 ^15^. The World Hepatitis Alliance’s call to “find the missing millions” reflects the gap in screening success. The increase in new HCV infection among young adults requires a shift in screening strategies. In some US states, young adults with HCV outnumber baby boomers ^16^. In some US states, young adults with HCV outnumber baby boomers ^16^ ( Figure 2). This raises the question of why universal one-time screening of adults is not adopted. By way of comparison, the USPSTF recommends HIV screening for all adults who are 15 to 65 years old, yet the infection burden for HIV is lower than HCV and HCV-associated mortality is higher than HIV in the US ^17^. Decision-analysis finds that one-time screening of all 15- to 30-year-olds is cost-effective if the prevalence of persons who inject drugs (PWIDs) in the cohort is more than 0.59% ^18^. Similar cost-effectiveness modeling could assist in expanding testing recommendations to capture those who remain infected but unaware of their HCV infection.

**Table 1.** Screening for hepatitis C virus.

Current recommendations	Expanded screening options
**Demographic-based** • Born 1945––1965 tested once • All pregnant women * **Risk-based** • Ever injected drugs • Received clotting factor concentrates before 1987 • Ever on long-term hemodialysis • Persistently abnormal alanine aminotransferase (ALT) levels • HIV infection • Transfusion of blood or blood components or an organ transplant before July 1992 • Health-care, emergency medical, and public safety workers after needle sticks, sharps, or mucosal exposures to hepatitis C virus (HCV)-positive blood • Children born to HCV-positive women	**High-prevalence groups** Universal (or opt-out) screening • Emergency/Acute care (select countries) • Prisons/Jails • Sexually transmitted disease (STD) clinics • Opioid substitution therapy (OST) clinics • Needle exchange programs • Homeless shelters
	**Universal screening of all adults from 18 to** **40 years of age** • Repeat screening per risk profile

*AASLD-IDSA HCV guidance recommendation ^34^

In the US and most countries, screening is performed primarily by primary care physicians (PCPs) and innovative strategies to maximize adherence to screening recommendations have been sought. Use of electronic reminders and best practice alerts have yielded excellent results ^19, 20^. In one study implementing electronic medical record (EMR) prompts for PCPs to perform HCV screening of baby boomers who lacked an anti-HCV result, rates of screening increased from 7 to 72% within a year of implementation ^20^. Of those diagnosed, 20% had advanced liver disease. Other studies have shown that EMR-based birth cohort screening both in the inpatient and outpatient settings is 2.6 to 8 times more effective than risk-based screening ^21, 22^ but that strategies to improve outcomes along the testing-to-care continuum were needed. HCV care coordinators and internal HCV “champions” within a practice or health-care system are useful in ensuring that those who test positive continue along the treatment cascade ^21, 23, 24^. Inclusion of HCV screening as a Medicaid quality metric would be anticipated to improve adherence to screening recommendations.

**Figure 3.**
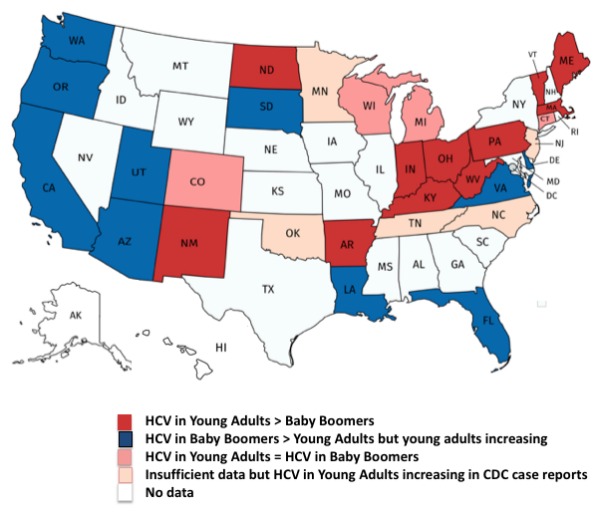
Hepatitis C virus (HCV) cases in young adults versus baby boomers in the US. To date, baby boomers have been the birth cohort with the highest prevalence of HCV infection. However, as cases related to the opioid epidemic increase among young adults, this is changing. Via state-level reports of HCV cases, the seroprevalence of HCV among baby boomers from 50 to 69 years of age was compared with those of young adults from 20 to 39 years of age, and the 2016 US Census population was used to determine the proportion of the population which was HCV-positive in each age category. Eleven states had higher rates of HCV in young adults than baby boomers, and four additional states had roughly equivalent numbers of young adults and baby boomers ^16^. CDC, Centers for Disease Control and Prevention.

For persons who are not consistently engaged with primary care, other testing opportunities need to be explored. Testing in emergency room departments, retail pharmacies, sexually transmitted disease clinics, and prenatal clinics has been proposed. Although these settings appear feasible for screening, completing the follow-up steps in the cascade of care ( Figure 3) is suboptimal ^25, 26^. Screening for HCV among PWIDs requires specific attention. Fueled in large part by the epidemic of opioid prescription and heroin use in the US, a doubling in rates of incident HCV infections occurred among adults from 20 to 40 years old from 2006 to 2012 ^27^. Point-of-care HCV testing using finger-stick whole-blood, dried blood spots, oral fluids, or venipuncture-based testing has been associated with increase testing and linkage to care among this population ^28, 29^. However, most point-of-care tests measure HCV antibody and require a venipuncture to assess for HCV viremia. This two-step process can lead to losses in care, especially as PWIDs may lack easy venous access. Advances in point-of-care tests of viremia (rather than antibody) provide unique opportunities to streamline care, especially in less traditional settings. In an Australian study conducted at drug and alcohol clinics, among homeless, and in needle and syringe programs, point-of-care testing with finger-stick capillary whole-blood samples (Xpert® HCV Viral Load test) was both sensitive and specific in identifying viremic (active infection) persons ^30^.

Another unique setting for HCV testing is in the prenatal clinic. Again, likely related to the “epidemic” of opioid and other drug use among those under age 40, the number of newly diagnosed cases of HCV among pregnant women increased by 89% from 2009 to 2014 ^32^. Currently, risk-based rather than universal screening is endorsed by the obstetrical societies ^33^, but this likely fails to identify infected women ^34^ because of either undisclosed risk factors by pregnant women or lack of inquiry regarding risk factors by providers. Recently, the American Association for the Study of Liver Diseases–Infectious Diseases Society of America (AASLD-IDSA) HCV guidance added a recommendation to screen all pregnant women for HCV ^35^. Identification of pregnant women with HCV provides an opportunity to plan HCV treatment post-partum with the intent to eliminate risk of vertical transmission for future pregnancies. Moreover, with mother-to-child transmission of HCV occurring in 5% of HCV-positive mothers, screening for HCV infection allows appropriate peripartum management and testing of infants ^36^.

Persons intersecting with the judicial system have a high prevalence of HCV, and rates among inmates vary from 10 to 40% ^37^. Screening approaches vary, but universal and opt-out strategies are best. In a study from the Dallas County (Texas) jail, uptake of testing increased from 13 to 81% when an opt-out rather than opt-in approach was used ^38^; 17% of inmates were HCV-positive. Cost-effective analysis shows that a universal opt-out HCV testing approach in prisons would decrease the number of new HCV infections and reduce liver-related deaths from HCV ^39^. In summary, to close that gap on under-diagnosis, both better application of the current guidelines for screening (that is, risk- and cohort-based) and expanded guidance for universal screening in selected, high-prevalence settings are needed.

## Under-treatment: more than just the cost

The impact of drug costs on access to HCV treatment is undeniable. The first approved DAA, sofosbuvir, had a list price in the US of $1000 per pill and all subsequent sofosbuvir-inclusive regimens have been higher, and the typical drug cost for a standard 12 weeks of ledipasvir-sofosbuvir is $94,500 ^40^. These initial prices resulted in practices to restrict access for many and have persisted even as drug costs have declined. For example, one of the most recently approved drug combinations, glecaprevir-pibrentasvir, costs only $26,400 for an eight-week course ^40^. Indeed, with rebates and discounting, the differences in costs between the different drug regimens are likely less than that suggested by wholesale list prices, so the competition among DAA manufacturers has helped to reduce the “cost per cure” and contributed to an increase in access to treatment. However, even among those who are insured, other costs may lead to less prescribing, as shown in a retrospective study from a large integrated health-care plan in California. In that study, higher maximum annual out-of-pocket health-care costs and having Medicare or Medicaid were associated with a 10 to 30% lower likelihood of initiating HCV treatment compared with patients having private health insurance ^41^. Globally, drug costs are a huge barrier to the elimination agenda, especially for middle- to low-income countries with a high prevalence of HCV infection. Some countries with high burden and limited resources have received substantial price reductions (up to 99%, for example, in Egypt). Approved DAAs have been included in the WHO’s Model List of Essential Medications to facilitate high-level drug price negotiations in countries that have national drug plans. More creative financial solutions will likely be necessary ^42^.

**Figure 4.**
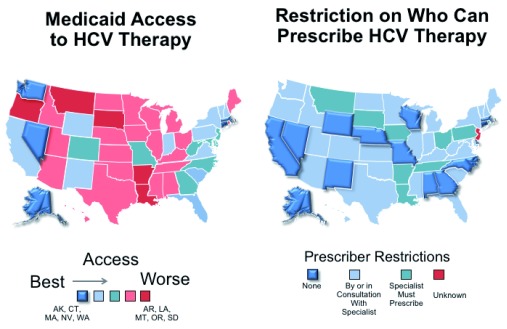
Rating of US states in terms of access to hepatitis C virus (HCV) treatment, 2017. A report from the National Viral Hepatitis Roundtable and Center for Health, Law and Policy Innovation at Harvard Law School found that most Medicaid programs restrict access to HCV treatment. More than half the programs received a “D” or “F” rating, indicating that severe restrictions to HCV therapy exist. In addition, restrictions on who can prescribe HCV treatment exist in all but 14 states. Adapted from sources: https://stateofhepc.org/wp-content/uploads/2017/10/State-of-Access-Infographic.pdf and https://stateofhepc.org/wp-content/uploads/2017/10/Prescriber-Infographic.pdf.

In the US, patients covered by Medicaid have experienced higher rates of treatment denial and have restrictions on who can prescribe DAA therapy that further contribute to reduced access to treatment ^43, 44^. Limiting treatment to only those with advanced fibrosis; requiring abstinence from alcohol, cannabis, and drugs for periods up to one year pre-treatment; and requiring the HCV treater to be a specialist are among the most frequently applied barriers. Restrictions based on recent drug use result in the inability to cure those with the highest risk for transmission. Restrictions based on alcohol use mean that patients at higher risk of liver disease progression and premature death are not receiving treatment. The National Viral Hepatitis Roundtable and the Center for Health Law and Policy Innovation highlighted the tremendous disparities across the US and developed a report card to showcase the best and worst states in terms of HCV access to treatment ( Figure 4) ^44^. Alaska, Connecticut, Massachusetts, Nevada, and Washington have no restrictions (grade A), but more than 50% of the states received a grade of D or F. The most restrictive states were Arkansas, Louisiana, Montana, Oregon, and South Dakota. As detailed below, there is no medical justification for these restrictions.

Restricting treatment to only those with advanced fibrosis ignores the substantial morbidity associated with chronic infection, including chronic fatigue ^45^, and the extrahepatic risks, such as diabetes or lymphoma ^45^. Indeed, cure has been shown to improve physical and mental domains, including work productivity, in HCV-infected persons with minimal or mild fibrosis ^46^. Additionally, treatment is shorter (and therefore cheaper) if carried out before the stage of cirrhosis. Finally, HCV cure before the development of advanced fibrosis eliminates the risk of future liver cancer whereas HCV eradication at the cirrhosis stage reduces but does not eliminate future liver cancer risk. For all of these reasons, treatment of HCV, regardless of stage, is the best strategy.

Treatment for persons who inject drugs is a high priority because of both the high burden of infection and the potential to transmit to others. The success of treating PWIDs is well established ^47^. In the recent SIMPLIFY trial, 103 persons with recent injection drug use (74% injected in the past month) received treatment with sofosbuvir-velpatasvir for 12 weeks and 94% achieved HCV cure with no virologic failures ^48^. Those with prior and current drug use, those on opiate substitution therapy, and those not on opiate substitution therapy had similar rates of cure with DAA therapy ^47, 48^. Modeling of treatment in populations of PWIDs highlights the need for prevention measures (safe syringe programs, opiate substitution programs, and safe injection houses) concurrent with HCV treatment.

Traditionally, HCV treatment was provided by specialists. This was justified in the peginterferon and ribavirin era when treatment was complex, associated with frequent side effects, and lengthy (24 to more than 48 weeks). Additionally, because treatment was frequently unsuccessful, many patients ultimately needed the specialist’s management of liver complications. But those days are gone as current HCV therapy is once-a-day dosing for 8 to 12 weeks and there are few if any side effects, yielding a cure in ≥95% ^35^. The simplicity of current DAA therapy allows PCPs to move into the primary position for treatment. Models of care that triage patients on the basis of severity of disease, with PCPs treating those without advanced fibrosis and specialists treating more complex patients, are effective ^49^. Studies comparing adherence and rates of cure in patients treated by front-line providers—nurse practitioners (NPs) and PCPs—highlight their success as HCV treaters. In the ASCEND study, NPs and PCPs new to HCV care were provided with three hours of intensive didactic training on HCV and its management and then 600 genotype 1 treatment-naïve patients with compensated liver disease were assigned to treatment with ledipasvir-sofosbuvir via an NP, PCP, or specialist. Cure rates were comparable across all provider types: NPs, 89.3%; PCPs, 86.9%; and specialists, 83.8% ^50^. Moreover, adherence to follow-up visits was higher with NPs and PCPs than with specialists! Specialists remain important not only in managing patients with more advanced liver disease or complex comorbidities but as educators and back-up support to PCPs engaged in HCV screening and treatment.

Project Expanding Capacity for Health Outcomes (ECHO), pioneered by the University of New Mexico, is a tele-health model that partners PCPs/NPs (spokes) with specialists (hub) to provide complex and comprehensive care ^51^. Using both didactic and case-based learning, PCPs are supported via videoconferencing to treat patients locally. This educational model was shown to yield outcomes as good as or better than those of specialists, and PCPs demonstrated increased knowledge and confidence in HCV care over time ^52^. In 2011, the Veterans Administration (VA) introduced the Project ECHO model nationally and they recently shared their initial positive results, and 21% of PCPs participated in at least one VA-ECHO undertaking treatment compared with only 2.5% among PCPs who did not attend ECHO ^53^. In California, Project ECHO was launched in 2016 and has successfully on-boarded PCPs/NPs across rural Northern and Central California to treat HCV ^54^. The challenge with these tele-mentoring models is that these activities are not reimbursed; rather, both specialist and PCPs donate their time to participate and to better serve their patients. However, for this to be a sustainable model, provider time to participate in Project ECHO needs to be compensated. In 2016, the US Senate passed the ECHO Act (S.2873), which required studies of the opportunities to use technology-enabled collaborative learning and capacity-building models for management of chronic diseases, including HCV. The results will hopefully provide a platform to advance partnerships between PCPs and specialists in caring for patients with HCV.

In elimination goals, prevention of new infections is as important as treating those already infected. In the US, the key focus for prevention is among PWIDs. Experts emphasize the need for integrated care—providing HCV treatment, addiction services, and general medical care—under “one roof” ^55^. Coupling HCV treatment with drug use counselling or opioid substitution therapy (OST) is important in reducing the likelihood of HCV reinfection. In the ANCHOR study, persons actively engaged in drug use were offered HCV treatment as well as OST. Of the 90 patients treated, 30% were on OST at baseline and 43% initiated OST during HCV treatment and 24% did not. Those on OST were more adherent to HCV visits and reported lower high-risk behaviors ^56^. Other models of care include directly observed HCV therapy and peer-based models ^57^. Innovation in this area of care delivery is essential to achieve elimination goals which include reducing incident infections by 90% by 2030.

## Concluding remarks

An estimated 189 million persons have been infected with HCV, and new infections are added to the global infection burden daily. The WHO’s call for HCV elimination requires each country to look critically at its cascade of care and develop strategies to amplify performance at each step. In the US, there is still much to be accomplished. Identifying those who are infected may require a broader screening mandate. One-time screening of all adults and repeat screening in at-risk groups such as those who inject drugs would get us closer to the goal. Management of HCV should increasingly be under the care of PCPs, and the expectation is that only those patients with advanced disease or who fail first-line therapy would need to be triaged to specialists. Specialists remain critical within these new models of care both to manage complex patients and to support and enable treatment among PCPs. Finally, drug costs remain a barrier in the US, as in many countries, and continued advocacy to reduce drug costs and remove the barriers imposed by insurers to prevent patient access to curative therapies is essential. Clinicians, researchers, public health experts, and advocacy groups all play a key role in the future of HCV elimination—it is an exciting but challenging opportunity!

## Abbreviations

DAA, direct-acting antiviral; EMR, electronic medical record; HCV, hepatitis C virus; NP, nurse practitioner; OST, opioid substitution therapy; PCP, primary care physician; PWID, person who injects drugs; USPSTF, US Preventive Services Task Force; VA, Veterans Administration; WHO, World Health Organization
